# The Development of a Competency Assessment Standard for General Practitioners in China

**DOI:** 10.3389/fpubh.2020.00023

**Published:** 2020-02-20

**Authors:** Xin Rao, Jinming Lai, Hua Wu, Yang Li, Xingzhi Xu, Colette Joy Browning, Shane Andrew Thomas

**Affiliations:** ^1^International Primary Health Care Research Institute, Shenzhen, China; ^2^Health and Family Planning Capacity Building and Continuing Education Center of Shenzhen Municipality, Shenzhen, China; ^3^Research School in Population Health, Australian National University, Canberra, ACT, Australia; ^4^School of Nursing and Healthcare Professions, Federation University, Ballarat, VIC, Australia

**Keywords:** competency standard, curriculum, general practitioners, China, GP training

## Abstract

This paper describes the development of a competency assessment standard for General Practitioners in Shenzhen, China. The standard is to be used for developing and delivering the training curriculum for General Practitioners and to enable rigorous assessment of the mastery of the standards by GP trainees. The requirement for the training of General Practitioners in China is mandated by government policy requires an international standard curriculum to meet the needs of patients and the community. A modified Delphi process was employed to arrive at a curriculum consensus. An expert panel and 14 expert working groups derived from the expert panel were established to review and evaluate national and international competency standards for General Practice and develop a set of standards, through a modified Delphi methodology. Forty three experts were involved in the project. The project resulted in a detailed curriculum statement. The curriculum was then used in 2017 and 2018 where pilot examinations of GP trainees (*n* = 298 and *n* = 315, respectively) were conducted to assess the trainee's competencies against the Standards. The examination included two modules, a written test (Module A) and a practical test (Module B). The success rate for participants was relatively low with the majority not successfully completing the assessments. The assessments will be further refined in subsequent work. The project achieved its goal of developing a rigorously evaluated standard to support clinical practice and the training and assessment of GPs.

## Introduction

As outlined in other papers by our team (1) in this special issue and by other commentators, the training of large numbers of high quality Chinese General Practice doctors is fundamental to China's efforts to improve its health system (2, 3). China has set ambitious targets for the growth in its General Practice medical workforce (4). This paper describes the development and pilot trial of competency standards for General Practice medical training in Shenzhen, China by the Health and Family Planning Capacity Building and Continuing Education Center of Shenzhen Municipality.

A key consideration in ensuring high quality service delivery by Chinese General Practice doctors is the development and implementation of rigorous clinical practice standards reinforced by a well-designed and executed training curriculum (5, 6). Recently, some commentators have addressed the issue of development of Chinese clinical practice standards and there has been some criticism of the quality of the standards development methodologies employed and the subsequent quality of the standards derived from them (7–9). The criticism has centered on the failure in many studies to satisfactorily address conflict of interest considerations and lack of reliance upon strong evidence tools such as systematic reviews to inform the content of the standards. International protocols for the development of standards particularly emphasize these two issues as central to the quality and credibility of high-quality clinical standards (10, 11).

In order to meet the needs of the Chinese people and indeed people of any nationality, rigorous, and high-quality approaches are required to ensure that doctors are well- trained and have access to evidence-based clinical standards.

The development of medical and nursing curriculums and clinical standards has often employed variants of Delphi technique as a means of rigorously developing agreed positions (12–15). Delphi procedures especially for curriculum development vary widely in their implementation with modifications from the original specification. We employed the same modifications employed by other curriculum developers who used this technique namely extensive discussion of content. Because a curriculum is expandable requests for additional content could be accommodated in most instances. We also had the advantage of extensive prior analysis of international curriculums and national and local epidemiology to guide the discussions. The Delphi technique, when properly applied, permits the structured management of conflict of interest issues and requires a systematic approach to the resolution of potential differences in opinions about the matters under discussion. However, the Delphi technique itself is not without some criticisms (16, 17) relating mostly to inconsistency and variability in the application of protocols. We would suggest that this may relate to the asking of answerable research questions for which there is adequate empirical evidence combined with the use of standardized and rigorous evidence evaluation protocols.

Globally, there has recently been a strong focus on the development of high-quality General Practice curriculum and practice standards (18–20). The content of international standards for General Practice/Family Medicine curriculums is, of course, an important focus, and guide for the development of the General Practice/Family Medicine discipline in China. However, each country has its own unique cultural, epidemiological and demographic circumstances (21). The curriculum and practice standards for one country do not necessarily wholly relate to the needs of another. The global burden of disease studies (22, 23) amply illustrate the variability in such circumstances between China and other countries. Sources such as the country reports from the WHO World Health Organization China country assessment report on aging and health (24) provide detailed data about the Chinese patterns of burden of disease that require attention in the Chinese medical curriculum. In Europe, members of the European Union have nevertheless made some efforts to harmonize the core content of General Practice training curriculums throughout the participant union countries. The Council of the European Academy of Teachers in General Practice and Family Medicine (EURACT) set themselves the task of specifying the common core content for a short clerkship in General Practice (25). The Council comprises the national representatives of EURACT. A Delphi consultation technique was used to derive an agreed list of 15 core areas for undergraduate General Practice/Family Medicine medical education curriculums.

In view of the strong emphasis placed by the Chinese government upon the necessity for rapid expansion and upgrade of general practice and the primary health care system, there is a strong awareness of the need for the development of modern, evidence-based general practice curriculums to ensure that the practitioners have the necessary skills and knowledge to deliver high quality care.

The goal of this research was to develop and trial rigorous evidence-based competency standards for General Practice medical training in Shenzhen, China. The research sub- questions that were specifically addressed to achieve this goal were:

1. What GP competency standards currently exist and what is their content?
2. What are the key patient groups in Shenzhen/ China?
3. What are the patients' health care needs?
4. What are the key clinical skills required for General Practitioners to address these needs?
5. What assessment tools should be used to test mastery of the knowledge and skills required to achieve the GP standards amongst trainee practitioners?

## Methods

### Expert Participants (*n* = 43)

The development of the curriculum assessment standards in this project was led by the Standards Unit within the Shenzhen Health and Family Planning Commission, the Shenzhen Health and Family Planning Capacity Building and Continuing Education Center. An expert panel and 14 groups comprising 43 expert participants were established to review and evaluate national and international competency standards for General Practice and develop a set of standards, through a modified Delphi methodology, to be applied in the training and assessment of General Practitioners in Shenzhen, China.

The experts were representatives from a range of local agencies and international experts including the Shenzhen Health and Family Planning Commission, the Shenzhen Health and Family Planning Capacity Building and Continuing Education Center, the Shenzhen International General Practice and Community Health Service Center, the Shenzhen Health Bureau General Practitioners Branch, the Shenzhen Hospital of Peking University, the General Practice Department of Shenzhen Hospital of Hong Kong University, and the US- based International Primary Care Education Alliance (IPCEA) and professors from Monash university and Australian National University. The experts had substantial clinical, teaching, curriculum development and management experience in primary care. The same 43 participants participated in all rounds of the study. At the commencement of the study agreement to participate in all rounds was obtained from the participants.

The foundation for the evidence base used in the Delphi methodology employed in this project was the NICE and NHMRC guideline protocols which emphasize:

- Forming the questions
- Deciding what evidence to include
- Identifying the evidence
- Selecting appropriate studies and documents
- Synthesizing evidence
- Assessing risk of bias
- Assessing certainty of evidence
- Documenting the evidence and final decisions.

The consultation protocols specified in these standards documents were followed meticulously. All decisions were documented in detail and provided to participants for further review to ensure that all matters had been fairly and inclusively dealt with. There was a very high commonality of views on all matters because of the evidence-based approach employed in the development process. For example, the epidemiology, disease burden, and demography of the community are facts informed by evidence for which detailed evaluation and evidence grading protocols were employed. The health needs of the community are knowable through this evidence. Such evidence based approaches provide additional rigor and certainty in the identification, analysis, and review of evidence to inform the consensus process.

### Trainee Trial Participants (*n* = 613 Participants)

As outlined below, two rounds of trials of the standards using examinations in the form of Objective Structured Clinical Examinations (OSCEs) were conducted (Rounds 3 and 4). In Round 3 the first pilot of the standards was conducted. The pilot involved an examination of 298 GP trainees seeking GP accreditation enrolled in the Shenzhen Health and Family Planning Capacity Building and Continuing Education Center. In Round 4 a second pilot involving 315 GP trainees seeking GP accreditation was conducted.

### Project Process and Methodology

**Figure 1:**
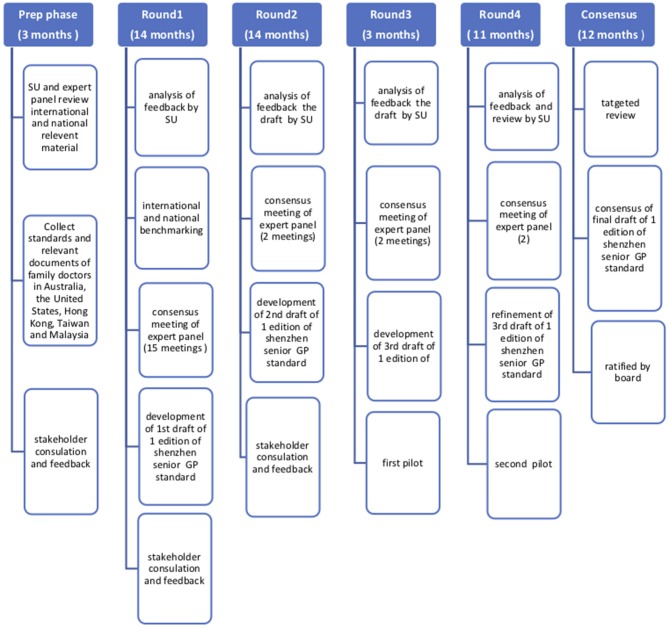
Flowchart depicting the projects steps and activities.

An extensive consultation process following modified Delphi methodology was undertaken in the project. Figure 1 summarizes the processes followed in the development of the Shenzhen GP training and assessment standards:

Each of the phases is now described. All meetings were face to face.

### Preparatory Phase (3 Months)—Analysis of Existing Local and International GP Standards

In the preparatory phase the activities focussed on the research question “What GP competency standards currently exist and what is their content?” The Standards Unit and the expert panel members reviewed GP competency standards from a range of Chinese and international bodies. These standards included those developed by:

- The Royal Australian College of General Practitioners (RACGP) (26, 27)
- The UK Royal College of General Practitioners (RCGP) (28)
- The Accreditation Council for General Medical Education Family Medicine Milestone Project (ACGME) (29)
- The Shenzhen Health and Family Planning Capacity Building and Continuing Education Center
- The World Organization of National Colleges, Academies and Academic Associations of General Practitioners/Family Physicians (WONCA) (30).

It should be noted that these documents are quite voluminous. For example, the RACGP document is 85 single spaced pages and the UK document is 98 single spaced pages. The detailed analysis of their overlapping and differential content is provided in supporting documentation available from the corresponding and first authors. The content analysis informed, but did not substitute for, the expert judgments of the 43 expert participants. A detailed questionnaire developed by the Standards Unit was circulated to 43 experts who were asked to assess the suitability of the content of the standards for General Practice in China. The results of the questionnaire guided the subsequent face to face discussions.

### Round 1—Identification of Key Patient Groups, Health Care Needs, and Associated Clinical Skills Required to Treat Them (14 Months)

In Round 1 of the process the activities focussed on the questions “What are the key patient groups in Shenzhen?” “What are their health care needs?” “What are the key clinical skills required for General Practitioners to address these needs?” The activities in this phase included a review of the local international and local GP standards. Fifteen face to face meetings of the expert groups were held to arrive at an initial draft of the first component of the standard. The component of the standard that was achieved through this round was the general consulting skills and knowledge domains that need to be held by GPs.

### Round 2—Development of Standards for Key Patient Groups, Common Symptoms, and Required Clinical Skills (14 Months)

The research questions addressed by this phase were “What are the key patient groups, medical issues, and basic skills that graduate GPs should be able to competently address?” This round focused on the development of a list of the top ten priority patient groups to be seen by GPs in Shenzhen. Local, national and international administrative and research data sets were interrogated to identify common symptoms, common diseases and severe and critical injuries within the Shenzhen community and how these related to other local and international jurisdictions (i.e., epidemiological benchmarking). The demographic and epidemiological data were also used to inform consideration of the basic clinical skills needed to prevent and treat the prevalent conditions. A draft list of key symptoms, conditions and basic operational skills was developed. Forty-three experts evaluated these domains which were developed through expert consensus (Draft 2 of the standards). Draft 2 of the standards comprised the general consulting skills and knowledge domains, the ten priority patient groups, key symptoms, severe conditions, and basic clinical skills and key symptoms. Two full panel face to face meetings with preparatory document review were conducted involving all the expert participants.

### Round 3—Development and First Trial of a Tool to Assess Mastery of the GP Standards by GP Trainees (3 Months)

In Round 3 the research aim addressed was “What written and practice modules in the form of Objective Structured Clinical Examinations (OSCEs) (31) should be used to test mastery of the knowledge and skills required to achieve the GP standards amongst trainee practitioners.” The expert panel rated the domains from Draft 2 and provided further feedback. Based on this feedback, Draft 3 of the GP standards was compiled by the supporting Standards Unit. In this round the first pilot of the GP standards was conducted. The pilot involved an examination of 298 GP trainees enrolled in the Shenzhen Health and Family Planning Capacity Building and Continuing Education Center. The examination included written and practice modules in the form of Objective Structured Clinical Examinations (OSCEs) examining general consulting skills and knowledge domains, the ten priority patient groups, key symptoms, severe conditions and basic clinical skills and key symptoms. Two full panel face to face meetings with preparatory document review were conducted involving all the expert participants.

### Round 4—Refinement and 2nd Trial of the Tool to Assess Mastery of the GP Standards by GP Trainees (11 Months)

In Round 4 the Expert Panel evaluated Draft 3 of the standards using the results of the first and a second pilot involving 315 GP trainees was conducted. The examination included written and practice modules in the form of Objective Structured Clinical Examinations (OSCEs). Two full panel face to face meetings with preparatory document review were conducted involving all the expert participants.

### Final Consensus Round—Finalization of the Standards, Curriculum, and Assessment Tools (12 Months)

The final Consensus round involved targeted review and refinement and final ratification by the Panel of the standards and the assessment tool to assess mastery of the GP standards by GP trainees. All documents were finalized in this phase and it involved extensive document review by the expert participants. The documents were submitted to government for final ratification. This was achieved and the program was then used to train the doctors. The November 10, 2017 edition of the Shenzhen Southern Metropolis Daily carried a lead article reporting the graduation of 24 Family doctors using the curriculum was announced. The doctors received senior accreditation and each received a cash reward of RMB 50,000 in recognition of their performance.

## Results and Discussion

The project ran for 47 months. The project timing was longer than planned but it included the development of the standards and two full trials of the tools developed to assess mastery of the standards by trainees. The key results for each round are now presented and discussed.

### Results of the Preparatory Round

In the Preparatory Round the Expert Panel participated in 15 workshops, reviewed the content of the existing local and international standards and developed new standards with a focus on China relevant standards and outcome indicators. From the Round 1 review and consultation process five domains of general consulting skills and knowledge were agreed as being core areas for Chinese General Practice (Draft 1 of the standards). Thus, the research questions “What are the key patient groups, medical issues, and basic skills that graduate GPs should be able to competently address?” were addressed.

Table 1 shows the final key domains of the 2019 Shenzhen General Practice Curriculum Standards derived from the expert panel consensus and consultations.

**Table 1:** Key domains in the 2019 shenzhen general practice curriculum standards key domains content.

**Key domains**	**Content**
General consulting skills and knowledge skills	Professional ethics and professional quality assurance
	Communication skills
	Clinical diagnosis and treatment in general practice
	People-centered, family-oriented and community-based health care
	Ability to utilize and coordinate health-resources
Ten priority patient groups:	1. Older people
	2. Women's health
	3. Men's health
	4. Patients with chronic and NCD health problems
	5. People with disabilities
	6. Migrant workers
	7. Multicultural residents
	8. Doctors
	9. Patients with critical illness and trauma
Key symptoms, conditions, severe, conditions and basic, clinical skills	40 common symptom clusters in the community
	78 common diseases in the community (Appendix 1)
	27 severe and critical conditions in the community (Appendix 2)
	33 basic operational skills in General Practice (Appendix 3)

The five domains were

1. Professional ethics and professional quality assurance,
2. Communication skills,
3. Clinical diagnosis and treatment in General Practice,
4. People-centered, family-oriented, and community-based health care,
5. Ability to utilize and coordinate health-resources.

These five domains were the headings used to group the standards content.

### Results for Round 2—Development of Standards for Key Priority Patient Groups, Common Symptoms, and Required Clinical Skills

The research questions for this phase were “What are the key patient groups, medical issues, and basic skills that graduate GPs should be able to competently address?” In Table 1, the 10 Key priority groups are listed along with the key symptoms, conditions, and basic clinical skills required to address them. Appendix 1 comprises the key symptoms identified in the research. Appendix 2 lists the severe and critical conditions in the Shenzhen community and Appendix 3 lists the basic operational skills identified in the Shenzhen community. These lists provided the basis for both the proposed curriculum and the assessment tools developed to assess appropriate competencies.

### Results for Rounds 3 and 4—Development and First and Second Trials of a Tool to Assess Mastery of the GP Standards by GP Trainees

In 2017 and 2018 pilot examinations of GP trainees (*n* = 298 and *n* = 315, respectively) were conducted to assess the trainee's competencies against the Standards. The examinations included two modules, a written test (Module A) and a practical test (Module B). Outcomes of the examinations are shown in Table 2. For Pilot 1 2017, the pass rate in Module A was 24% and for Module B it was 34%. In Pilot 2 in 2018, the pass rate for Module A was 14% and for Module B it was 48 per cent. Thus, the assessment modules were proven to be quite rigorous. It could be argued that the low pass rates were a function of poor quality teaching. This explanation is considered unlikely because the instructors were nationally and internationally accredited with significant and lengthy experience in accredited Chinese, US and Australian General Practice and Family Medicine programs. In the future the tools will be improved and refined with further use and development.

**Table 2:** Outcomes of the round 3 and round 4 pilot examinations.

**Pilot/Module**	**Pass rate**	**Difficulty co-efficient**	**Discriminant analysis**	**Reliability (Cronbach's alpha)**
**Pilot 1 2017** ***n=*** **298**				
Module A	0.24	0.55	0.24	0.65
Module B	0.34	0.54	0.33	0.60
**Pilot 2 2018** ***n*** **=** **315**				
Module A	0.14	0.41	0.31	0.65
Module B	0.48	0.53	0.46	0.87

### Study Strengths and Limitations

This study used a widely representative group of Chinese and international medical experts in General Practice and Primary Health Care, using a structured Delphi process to develop the General Practice standards specification. As outlined in the study description, the Delphi method is now a widely used method for generation of consensus statements about clinical standards and curriculum specifications although it does have some shortcomings. The process of curriculum generation has also been informed by rigorous review of other international curriculums and existing national and local Chinese curriculum standards. A rigorous iterative process has been employed in the refinement of the final curriculum statements.

The curriculum has been trialed in a large group of GP trainees (*n* = 613) and the assessment tools associated with the curriculum trial have been refined. The assessments have been found to be quite rigorous with high standards as reflected in the relatively low pass rate for those who sat the assessments.

The study limitations included the very substantial resources required to implement the project and the longer than expected project duration (initially a 3-year period was proposed but an additional year was necessary). The documented limitations of Delphi methods may apply to this project.

## Conclusions

This research involved a lengthy development process based upon Delphi procedures involving a large expert group of 43 participants. The curriculum that has resulted from the process has been tested and refined with two sizeable cohorts of trainees (in 2017, *n* = 298 and in 2018, *n* = 315). It will be further refined and developed as it is implemented as part of standard quality improvement processes. While the need for substantially increased numbers of General Practice doctors is recognized in policy and in resource allocation, it is important that the Chinese trainees when they graduate are able to meet internationally recognized standards of practice. An important aspect of this project is the development of standard curriculum and rigorous standardized assessment processes that meet local and national needs. That said, curriculums need to be dynamic and adapt to meet changing circumstances. In China, these circumstances include rapidly changing demography and strong growth in chronic illness and age-related conditions as well as meeting the needs of young people and men and women. We thank the many clinicians and researchers who assisted with this epic task.

## Data Availability Statement

All datasets generated/analyzed for this study are included in the article/Supplementary Material.

## Ethics Statement

Ethical review and approval was not required for the study on human participants in accordance with the local legislation and institutional requirements. The patients/participants provided their written informed consent to participate in this study.

## Author's Note

This paper provides an overview of a major program of activity undertaken in Shenzhen with the purpose of developing General Practice standards, an associated curriculum and assessment tools to assess mastery of the standards and clinical skills. The documentation of these components is voluminous. Queries concerning the availability of these documents and associated resources should be addressed to the corresponding author.

## Author Contributions

All authors participated in the design of the study and contributed to the drafting of the paper.

### Conflict of Interest

The authors declare that the research was conducted in the absence of any commercial or financial relationships that could be construed as a potential conflict of interest.
