# Supplementary material for: The Development of a Competency Assessment Standard for General Practitioners in China
Source: Front Public Health. 2020 Feb 20;8:23. doi: 10.3389/fpubh.2020.00023 (PMC7045360; doi:10.3389/fpubh.2020.00023)
Supplement: Supplementary file 1 [file Data_Sheet_1.docx]

**Appendix 1**

**Key symptoms identified in the Shenzhen General Practice curriculum**

1. fever
2. edema
3. fatigue
4. obesity
5. weight loss
6. headache
7. dizziness
8. cognitive disorder
9. sensory disturbance
10. epistaxis
11. tinnitus
12. eyes red with pain
13. hearing disorder
14. visual disturbance
15. cough
16. dyspnea
17. pharyngalgia
18. chest pain
19. palpitation
20. abdominal pain
21. vomiting
22. diarrhea
23. abdominal swelling
24. dyspepsia
25. hematochezia
26. hematuria
27. paruria
28. subcutaneous nodules
29. breast mass
30. abdominal mass
31. neck pain and shoulder pain
32. low back pain and leg pain
33. back pain, thoracic
34. Joint pain
35. Leukorrhea abnormality
36. abnormal vaginal bleeding
37. amenorrhea
38. baby crying
39. dysplasia
40. rash

# Appendix 2

**Severe and critical conditions in the community in the Shenzhen General Practice curriculum**

1. sudden cardiac arrest
2. acute airway obstruction
3. spontaneous pneumothorax4
4. hypertensive emergency
5. acute coronary syndrome
6. fateful arrhythmia
7. allergic shock、
8. hypovolemic shock and septic shock
9. upper gastrointestinal haemorrhage
10. infantile convulsion
11. toxicosis
12. heliosis
13. drowning
14. animal stings
15. acute abdomen
16. cerebral infarction、cerebral haemorrhage and TIA transient ischemic attack
17. multiple trauma
18. craniocerebral trauma
19. pneumothorax
20. pulmonary contusion
21. rib fracture
22. cervical spinal trauma
23. spinal trauma
24. paraplegia
25. limb fracture
26. hand trauma
27. burn injury

# Appendix 3

**Basic operational skills identified in the Shenzhen General Practice curriculum**

1. physical examination
2. sputum suction
3. oxygen treatment
4. electrocardiograph operation and electrocardiogram diagnosis
5. digital rectal examination
6. laboratory diagnosis
7. urethral catheterization
8. enema
9. various methods of injection
10. collection of specimens including feces, urine, sputum, blood)
11. children's growth and development assessment
12. the operation of CPR in children
13. aseptic operation
14. debridement and suture of small wounds
15. dressing and taking out stitches for various wounds
16. excision of superficial mass
17. incision and drainage of superficial abscess
18. the use of the speculum
19. vaginal discharge examination
20. cardiopulmonary resuscitation CPR)
21. electric defibrillation
22. the use of simple respirator
23. wound dressing, hemostasis and fix
24. tracheal intubation
25. paracentesis
26. skin biopsy
27. indications of cryopreservation and laser treatment
28. the use of vision examination and ophthalmoscope
29. treatment of eye washing
30. the use of nasal endoscopy and ear mirror
31. conventional sterilizing and isolating
32. doctor's self-protection such as hand hygiene)
33. commonly used psychological screening scales such as self-rating depression scale SDS), self-rating anxiety scale SAS), scale for dementia)
